# Unmasking the microbiome: the hidden role of gut bacteria in the pathogenesis of colorectal cancer and its prevention strategies

**DOI:** 10.37349/etat.2025.1002351

**Published:** 2025-11-24

**Authors:** Tallha W. Khawaja, Lei Zhao, Raiq Siddiq, Mohammad U. Ahmad, Caitlin P. Burns, Jacob M. Parker, Mark R. Wakefield, Yujiang Fang

**Affiliations:** IRCCS Istituto Romagnolo per lo Studio dei Tumori (IRST) “Dino Amadori”, Italy; ^1^Department of Microbiology, Immunology & Pathology, Des Moines University College of Osteopathic Medicine, West Des Moines, IA 50266, USA; ^2^Department of Respiratory Medicine, the 2nd People’s Hospital of Hefei and Hefei Hospital Affiliated to Anhui Medical University, Hefei 230011, Anhui, China; ^3^Department of Surgery, University of Missouri School of Medicine, Columbia, MO 65212, USA; ^4^Ellis Fischel Cancer Center, University of Missouri School of Medicine, Columbia, MO 65212, USA

**Keywords:** colorectal cancer, gut microbiota, dysbiosis, probiotics, fecal microbiota transplantation (FMT)

## Abstract

Colorectal cancer (CRC) is a significant global health problem, ranking as the third most common cancer and the second leading cause of cancer deaths in the world. The highest incidence of CRC is found in developed regions, thus underlining its characterization as a Western disease. Major risk factors for CRC include an unhealthy diet, lack of physical exercise, and cigarette smoking. The gut microbiota refers to the complex community of microorganisms inhabiting the digestive tract and plays a crucial role in the maintenance of host health and modulation of immune responses. Gut dysbiosis can be caused by poor diet and alcohol consumption, increasing CRC risk. Specific bacteria, such as *Fusobacterium nucleatum* and *Escherichia coli*, may have a close relationship with CRC development, while the beneficial bacteria are frequently depleted in CRC patients. This paper will discuss the mechanisms of colorectal carcinogenesis, focusing on the effects of bacterial genotoxins, immune evasion, inflammation, and diet. Additionally, it reviews preventative strategies including short-chain fatty acids (SCFAs), prebiotics, probiotics, synbiotic supplements, and the method of fecal microbiota transplantation (FMT), showing their potential to improve overall gut health and reduce the risk for CRC. Understanding these mechanisms and implementing specific preventative strategies could significantly enhance clinical interventions and reduce the global burden of CRC.

## Introduction

Colorectal cancer (CRC) is an emerging global health burden, characterized as the third most common kind of cancer, and is the second-leading cause of cancer-related death worldwide [1]. CRC is prevalent in highly developed countries, including the United States, Canada, and parts of Europe. By contrast, Africa and South Central Asia have a relatively low incidence of CRC, making it primarily a Western disease. CRC affects both men and women, though males generally have a slightly higher incidence rate [2]. The major lifestyle risk factors for CRC include diet, physical activity, and smoking. It is estimated that high processed red meat consumption and low fiber, saturated fat, calcium, and vitamin D intake confer an increased CRC risk, while regular physical activity and ideal weight can reduce CRC risk [3]. Moreover, genetic predispositions and environmental factors can increase the likelihood of CRC; regular screening and early detection may be necessary in people who have a family history of the disease [1, 3]. CRC can disrupt different parts of the colon and is classified as proximal (right-sided) or distal (left-sided). Distal CRC is more prevalent and can include the sigmoid colon or the rectum. This is contrasted to right-sided cancers, which have a poorer prognosis compared to other forms of CRC [4]. Understanding the various loci and mechanisms of CRC can contribute to the development of location-specific preventative strategies or treatment modalities.

The gut microbiota consists of a diverse community of microorganisms residing in the digestive tract that work in concert with the host. Its development begins at birth and is affected by things such as mode of delivery and breastfeeding, followed by environmental exposures. Once established, the gut microbiota plays a crucial role in maintaining host health, primarily residing in the colonic compartment, where it plays a vital role in digestion, metabolism, and immunological development [5]. It degrades complex carbohydrates, synthesizes essential vitamins, and modulates the gut barrier integrity [6, 7]. The gut microbiota can also modulate the immune system through the production of short-chain fatty acids (SCFAs) such as butyrate, which are the end products of dietary fiber fermentation by gut bacteria. Butyrate supports the immune system by promoting the differentiation and function of regulatory T cells (Tregs) [6–8]. Moreover, the interference of the gut microbiota with the immune system may further affect the innate immune response. Through interaction with pattern recognition receptors, like Toll-like receptors, the gut microbiota can modulate the activity of the immune system. This enhances the body’s ability to identify and respond to pathogens and can exert some pro-inflammatory effects [9]. These interactions provide insight that could lead to the development of targeted prevention and treatment strategies based on the objectives of restoring the gut microbiota and hence reducing the risk of CRC through the use of probiotics or dietary interventions.

Dysbiosis, which is an imbalance in the levels of different gut microbiota, can significantly increase the risk of CRC. Risk factors such as a poor diet and significant alcohol consumption have the ability to disrupt the gut microbiome function, promoting a cancer-conducive environment [10]. For instance, a diet high in processed red meats and low in fiber may change the levels of gut bacteria, while alcohol can directly exacerbate inflammation and increase the growth of harmful bacteria [11]. *Fusobacterium nucleatum*, an opportunistic pathogen, is frequently found at elevated levels in CRC patients and linked to poor prognosis. This bacterium promotes tumor growth, metastasis, and an inflammatory environment, making it a potential CRC biomarker [12]. Other bacteria associated with CRC include *Bacteroides fragilis*, *Escherichia coli*, *Enterococcus faecalis*, and *Streptococcus gallolyticus*, each contributing to disease progression through toxin production, chronic inflammation, and immune system modulation. Conversely, beneficial bacteria such as *Bifidobacterium*, *Lactobacillus*, *Enterococci*, and *Propionibacteria* are often depleted in CRC patients [7]. The benefits of the gut microbiota to the host’s physiology are vast, including nutrition, immune development, and host defense [13]. Some bacterial species present in the gut also have the ability to participate in the biosynthesis of several components, such as vitamin K and vitamin B [14]. As stated above, SCFAs are a byproduct of dietary fiber fermentation by the gut bacteria, which can ultimately play a role in the immune response and further prove the gut bacteria’s necessity for human function and well-being.

Comprehending the pathways in which gut dysbiosis contributes to the development of CRC facilitates the formulation of focused prevention interventions. Diet modification, supplementation with probiotics, prebiotics, synbiotics, SCFAs, and fecal microbiota transplantation (FMT) are approaches that restore a healthy gut microbiome, regulate immune responses, and reduce the risk of developing CRC. This review highlights the pathogenic mechanisms as well as the potential of these microbiota-centered interventions.

## Pathogenesis of CRC

**Figure 1:**
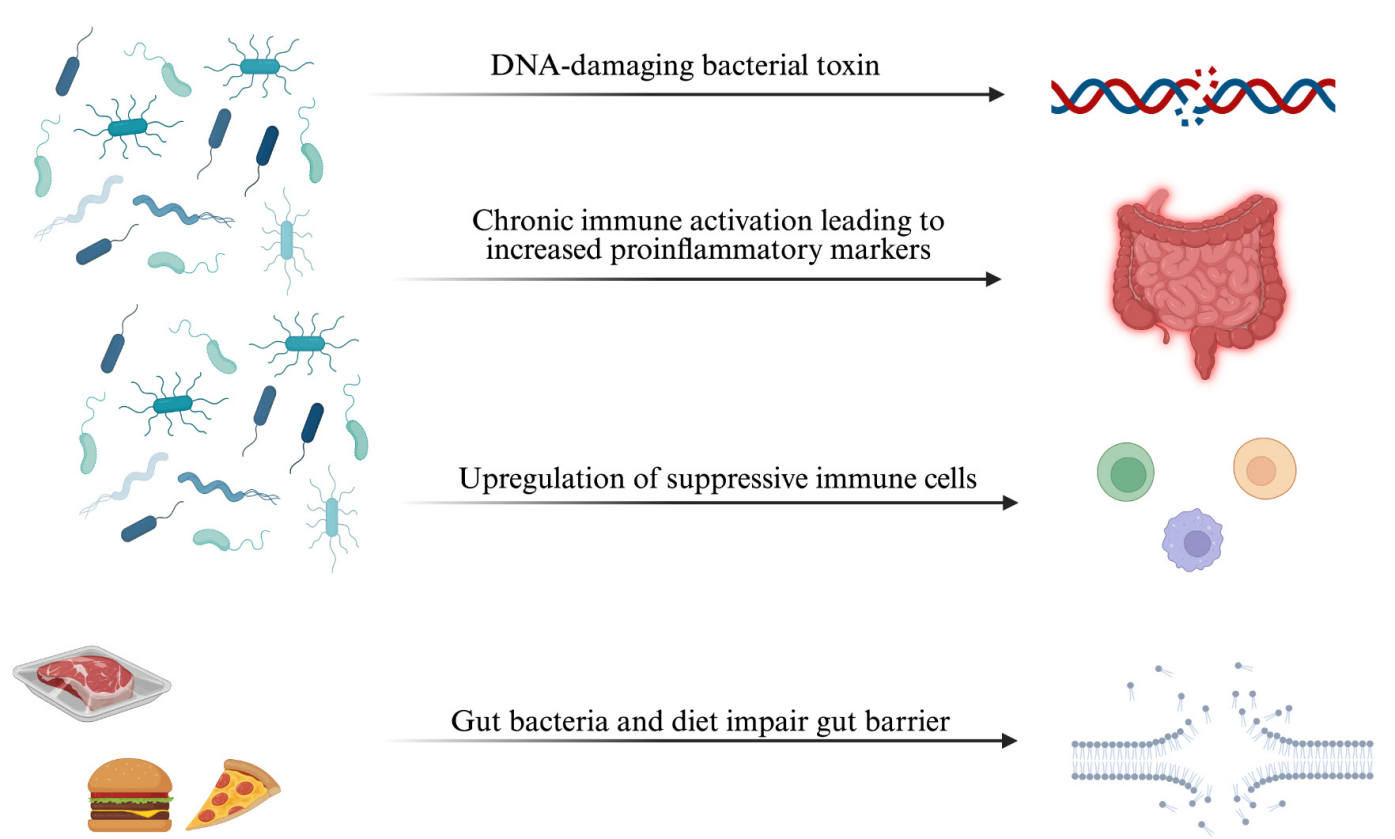
**Overview of gut microbiota-driven mechanisms in colorectal cancer.** DNA-damaging bacterial toxins induce genomic instability, while dysbiosis triggers inflammation and proinflammatory cytokine release. Gut microbes upregulate suppressive immune cells, and together with diet, disrupt gut barrier integrity—promoting tumor development. Created in BioRender. Khawaja, T. (2025) https://BioRender.com/c2nwdgb.

Although the specific pathways that lead to the development and progression of CRC are unknown, several variables have been identified as crucial. These variables consist of immunological evasion strategies, nutritional effects, chronic inflammation, and bacterial genotoxins. The effects of these strategies are visualized in Figure 1. We will provide further explanation about these recommended techniques in the upcoming sections.

Genotoxins derived from various bacteria play a critical role in the development and further progression of CRC. For instance, colibactin, produced by polyketide synthase-bearing *Escherichia coli* strains, and a cytolethal distending toxin produced by *Campylobacter jejuni*, have the ability to induce double-stranded breaks in the host cell DNA [15, 16]. These genotoxins induce a signaling cascade of DNA damage leading to persistent and sustained mitosis due to the inactivation of checkpoints, chromosomal aberrations, and an increased rate of genomic mutations, resulting in the development of various cancers [17]. According to the driver-passenger model, driver bacteria are a subpopulation of native intestinal bacteria that induce CRC by producing genotoxic compounds that damage the DNA in epithelial cells. These initial alterations in the tumor environment further select for the overgrowth of opportunistic bacteria, termed passenger bacteria [18]. The genotoxins produced by the gut microbiota constantly initiate DNA damage in host epithelial cells in synergy with chronic inflammation and other environmental factors within the gut microenvironment, facilitating CRC initiation and its further progression [19].

Immune evasion is a hallmark process in CRC progression, whereby tumors can evade the host’s defense mechanisms. *Fusobacterium nucleatum* has been shown to play an essential role in increasing myeloid-derived suppressor cells (MDSCs), CD11b+ cells, M2-like tumor-associated macrophages (M2 TAMs), and tumor-associated neutrophils (TANs). These cells act as negative regulators of antitumor immunity [20]. Furthermore, *Fusobacterium nucleatum* can produce an inhibitor protein that strongly inhibits the activity of human T-cells by causing cell cycle arrest at the G1 phase, creating an immunosuppressive environment favorable to tumor cell proliferation [21]. Another critical factor involved in immune evasion is the transcription factor SOX-17, which is not expressed in the adult normal intestinal epithelium but is re-expressed at the beginning of tumorigenesis. It acts by reducing the expression of the receptor for interferon-gamma (IFNGR1), dampening colon cancer cells’ ability to respond to this critical cytokine [22]. Because interferon-gamma (IFNγ) signaling is critical for a successful anticancer immune response, loss of this receptor pathway is associated with resistance to immune checkpoint blockade in patients [23]. The immunosuppressed environment initiated by these tumor cells not only allows for the escape of immune surveillance but also sets the stage for chronic inflammation, further promoting carcinogenesis.

Inflammation is another important process for CRC initiation and progression [24]. Gut pathogenic bacteria can activate several signaling pathways associated with IL-17 and NF-κB, and pattern recognition receptor-mediated pathways, resulting in inflammation. These pathways have critical roles in immune response and inflammation and often disrupt the gut barrier function, further fuelling the inflammatory process [25]. Specifically, *Bacteroides fragilis* initiates an inflammatory cascade involving activation of the IL-17R, NF-κB, and STAT3 signaling pathways in colonic epithelial cells, followed by carcinogenesis. The basis for this induction is chronic inflammation, which then provides an appropriate microenvironment for cancer [26].

Diet also has a vital role in the development of CRC. A high intake of red meat is associated with an increase in sulfur production, which is known to have possible carcinogenic action capable of damaging the colonic mucosa and favoring carcinogenesis [10]. Conversely, a decreased dietary fiber intake results in a reduced production of SCFAs, which are important in maintaining gut health and preventing inflammation. Eventually, reduced production of these SCFA will increase the risk of developing CRC [27]. Additionally, increased levels of secondary bile acids, such as deoxycholic acid, have been linked to increased CRC susceptibility in individuals adhering to a high-fat diet [28]. All these dietary factors will result in an environment conducive to cancer development, highlighting the importance of dietary choices in CRC prevention.

## Prevention and therapy strategies

Understanding the underlying processes of CRC carcinogenesis enables the implementation of specific prevention methods. These methods are designed to lessen the effects of negative factors created by the pathogenesis of CRC, boost the positive factors, and ultimately lower CRC risk. The importance of dietary components, probiotics, prebiotics, synbiotics, and FMT stands out among these approaches. These methods not only affect the gut microbiota composition but also aim to regulate the inflammatory and immune responses, leading to a more conducive environment for gut health and cancer prevention.

Among the different CRC prevention strategies, SCFA, specifically butyrate, plays a prominent role. Butyrate is a compound synthesized via the fermentation of dietary fibers by the gut microbiota and possesses many different anti-tumor activities [8]. It preserves the integrity of the colonic epithelium, which is crucial in preventing cancer development. It is also postulated that butyrate promotes healthy colonocyte turnover by providing an energy source, promoting cell differentiation, and growth [29]. Butyrate also enhances insulin sensitivity and lowers adiposity, potentially reducing CRC risk [30, 31]. Additionally, it has an anti-inflammatory action by blocking NF-κB activation, which plays a strong role in the inflammatory process during tumor development [32]. These protective effects can be promoted by increasing the consumption of fiber or by directly taking butyrate supplements. A dietary intake rich in fiber is essential in preventing CRC; other SCFAs, namely acetate and propionate, also play an essential role in gut health [33]. Acetate, the most abundant SCFA, serves as fuel for cells of the distal gut, modulates lipid metabolism, and regulates appetite [34]. Propionate inhibits cholesterol synthesis and exhibits anti-inflammatory properties [35]. Collectively, SCFAs provide a healthy gut environment, increase immune function, and reduce inflammation, further reducing the risk of CRC.

Probiotics, which are live microorganisms that provide health benefits, have also demonstrated promise in modulating the gut microbiota. This is done through promoting colon health and supporting the intestinal barrier function, thus making them relevant in the context of CRC [36]. As dysbiosis is connected to the development of CRC, probiotics play a critical role in preserving the equilibrium of the gut microbiome. In a noteworthy study, 60 patients following surgical CRC resection participated in a randomized, double-blind, placebo-controlled experiment to demonstrate these effects. In this group, 29 patients were given a probiotic powder containing 1 × 10^8^ CFU of *Bifidobacterium animalis* subsp. *lactis* (HY8002), 5 × 10^7^ CFU of *Lactobacillus casei* (HY2782), and 5 × 10^7^ CFU of *Lactobacillus plantarum* (HY7712) for four weeks, beginning one week prior to surgery, while the remaining 31 patients were given a placebo. Beneficial bacteria like *Bifidobacterium*, *Akkermansia*, *Parabacteroides*, *Veillonella*, and *Lactobacillus* were more prevalent in the therapy group. At the same time, there was a noticeable decrease in CRC-associated bacteria, such as *Porphyromonas*, *Fusobacterium*, *Alloprevotella*, and *Prevotella* [37].

This change in the gut microbiota composition suggests that probiotic supplements can improve the gut environment and potentially lower the CRC risk. Probiotics help maintain gut health by enhancing gut barrier function, mainly by upregulating or normalizing tight junction expression. This prevents the leakiness commonly found in CRC patients, which allows for microbial translocation [38]. Additionally, probiotics can induce apoptosis in CRC cells, eliminating malignant cells and halting further tumor growth. Furthermore, probiotics have anti-inflammatory qualities that help reduce gut inflammation [39]. Probiotics, therefore, form one of the many dietary strategies targeted at reducing CRC risk due to their capacity to modify the immune response, underpinning their role in cancer prevention [40]. Probiotics are helpful in maintaining gut health and offer protection from inflammation and carcinogenesis through their interactions that result in an increased number of good bacteria and a reduced amount of dangerous bacteria [37].

Prebiotics, or non-digestible food elements, selectively stimulate the growth and activity of beneficial intestinal bacteria, hence promoting gut health and reducing CRC risk in the host [41]. While not all dietary fiber may be considered a prebiotic, all prebiotics derive from dietary fiber [42]. To be labelled as a prebiotic, three criteria must be met: resistance to stomach acidity, hydrolysis by digestive enzymes, and resistance to upper gastrointestinal system absorption. It must also be fermented by intestinal microflora and specifically increase the growth and activity of beneficial gut bacteria [43]. Many foods, including soy beans, chicory root, oats, wheat, and cornstarch, naturally contain prebiotics [44]. These foods support the growth and nutrition of beneficial bacteria like *Lactobacillus* and *Bifidobacterium*, which are essential for a balanced gut microbiota [45]. One significant outcome of prebiotic fermentation is the synthesis of SCFAs, particularly butyrate. Apart from providing primary energy to colonocytes and maintaining the integrity of the colonic epithelium, butyrate also reduced colonic mucosal damage by decreasing certain serum inflammatory cytokines, including IL-6 and TNF-α [46].

The term “synbiotics” refers to the advantageous effects of combining the actions of both probiotics and prebiotics, offering the potential to significantly enhance gut health [47]. Prebiotics nourish probiotics, fostering a more robust and resilient gut microbiota. By enhancing the ratio of helpful bacteria and boosting the synthesis of SCFAs like butyrate, this combination improves the balance of beneficial bacteria, controls immune responses, and reduces inflammation [48]. Chronic inflammation increases the levels of IL-6, STAT3, NF-κB, PGE-2, COX-2, and TNF-α, promoting a pro-carcinogenic environment and CRC progression [49]. Synbiotics have been shown to reduce these inflammatory markers, mitigating their harmful effects. By decreasing IL-6, STAT3, NF-κB, PGE-2, COX-2, and TNF-α levels, combining the effects of prebiotics and probiotics helps lower inflammation and inhibit cancer-promoting pathways, offering a promising strategy for CRC prevention and treatment [50]. Using probiotics and prebiotics in tandem provides a comprehensive approach to preserving gut health and reducing CRC risk. Table 1 helps to summarize the status of prebiotics, probiotics, and synbiotics along with their potential contributions in CRC prevention.

**Table 1:** Effects of probiotics, prebiotics, and synbiotics on gut health.

**Intervention**	**Mechanism of action**	**Effect on gut microbiota**	**Impact on CRC prevention**
**Probiotics**	Inactivate carcinogens and regulate apoptosis and cell differentiation. Helps restore the intestinal barrier and reduce inflammation	↑ *Bifidobacterium*, *Akkermansia*, *Parabacteroides*, *Veillonella*, *Lactobacillus* ↓ *Porphyromonas*, *Fusobacterium*, *Alloprevotella*, *Prevotella*	Improves gut environment, enhances barrier integrity, induces apoptosis in CRC cells, and reduces inflammation
**Prebiotics**	Selectively helps to stimulate beneficial bacteria, and ferments to produce SCFAs (esp. butyrate)	↑ *Lactobacillus*, *Bifidobacterium*, SCFA production (butyrate)	Maintains colonic epithelium integrity, reduces IL-6 and TNF-α, lowers mucosal damage, improves immune balance
**Synbiotics**	Synergistic effects: prebiotics nourish probiotics; ↑ SCFA (butyrate) production; regulate immune signaling	Enhanced balance of beneficial bacteria; ↑ SCFA synthesis	Decrease IL-6, STAT3, NF-κB, PGE-2, COX-2, TNF-α; reduce chronic inflammation and inhibit cancer-promoting pathways

The table summarizes the mechanisms and preventive effects of probiotics, prebiotics, along synbiotics in CRC. It is based on current evidence in this study. ↑: indicates an increase; ↓: indicates a decrease. CRC: colorectal cancer; SCFAs: short-chain fatty acids.

**Figure 2:**
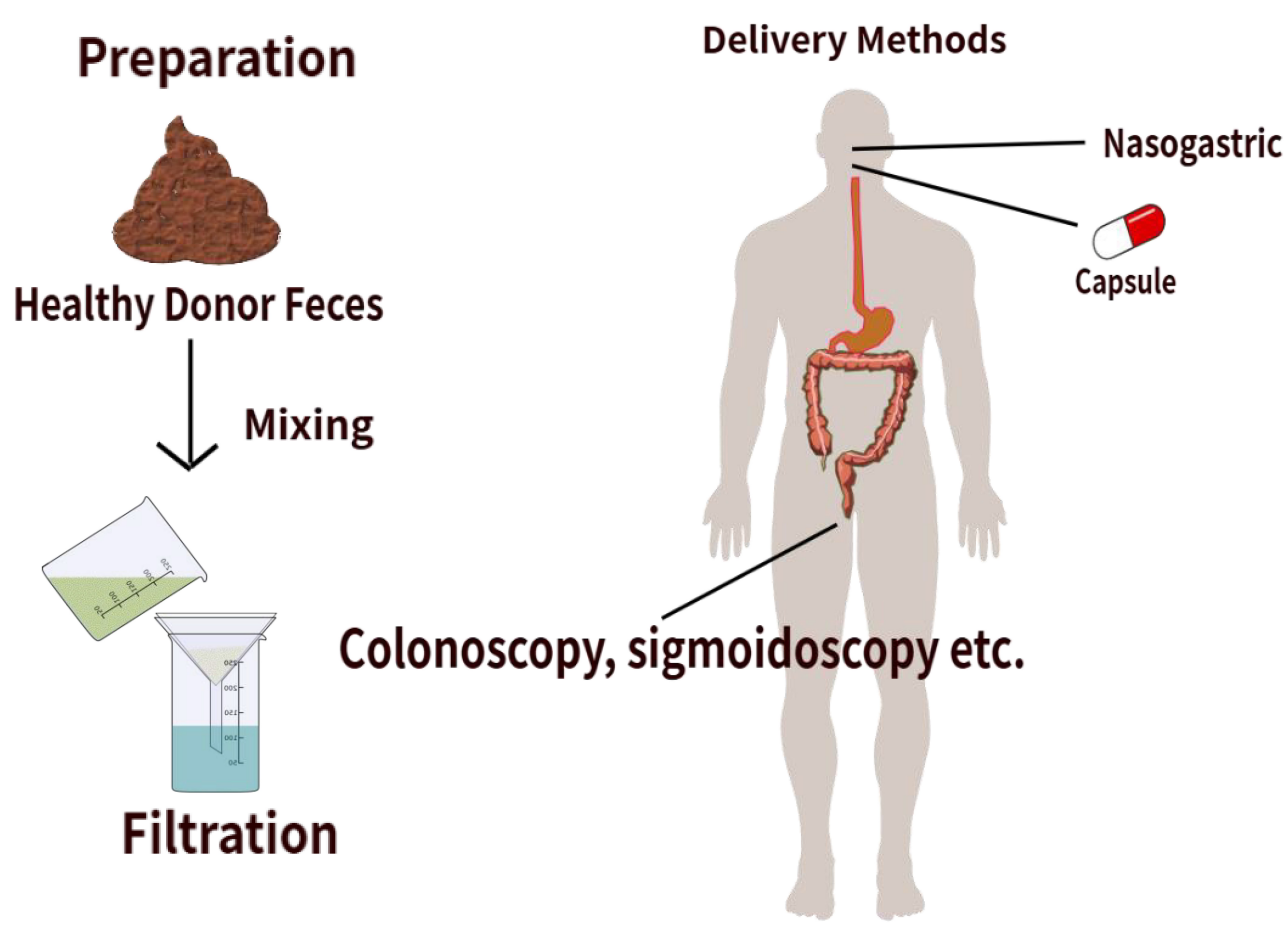
**FMT in colorectal cancer.** The FMT process collects fecal matter from a healthy donor. A process of mixing and filtering the material prepares a microbiota-rich suspension. The material is then delivered to the recipient either via lower or upper GI routes. This restores a balanced gut microbiota, reduces harmful bacteria, and increases beneficial species. FMT: fecal microbiota transplantation; GI: gastrointestinal.

FMT involves the transfer of fecal matter from a healthy donor into a recipient’s gastrointestinal tract to reestablish a well-balanced microbiota [51]. This can be visualized in Figure 2. There are a variety of delivery methods for FMT, including upper gastrointestinal administration via upper esophagogastroduodenoscopy, nasogastric, nasojejunal, and nasoduodenal tubes, or oral capsules. It can also be administered through the lower gastrointestinal route via colonoscopy, sigmoidoscopy, or an enema [52]. Evidence from preclinical mouse studies has shown that the use of FMT has beneficial effects on CRC. This was done by decreasing the inflammation and reducing the abundance of cancer-promoting bacteria. A recent study has shown that treatment with anti-PD-1 (4 × 200 µg) and FMT (4 × 5 × 10^9^ CFU) from healthy human donors improved survival and reduced tumor growth in CT26-tumor-bearing mice. This combined treatment increased the abundance of *Parabacteroides distasonis* and other beneficial bacteria while reducing the abundance of harmful bacteria, such as *Clostridium* sp. HGF2, *Enterococcus hirae*, *Dorea* sp. 52, and *Lactobacillus murinus*. Moreover, there was an increased abundance of particular Bacteroides species, such as *B*. *thetaiotaomicron* [53]. *B*. *thetaiotaomicron* maintains intestinal homeostasis by inducing dendritic cells through microbe-host crosstalk, ultimately suppressing CRC carcinogenesis via its metabolite, propionate [54, 55]. Increased levels of aspirin, a growth inhibitor of the CRC-associated bacterium *Fusobacterium nucleatum*, and higher levels of punicic acid, known for its antitumor activities, were also observed [53]. These results further support that combining FMT with immunotherapy could be a very promising approach for CRC treatment, although further clinical studies are needed to study the efficacy in humans.

## Conclusions

In summary, CRC remains an important health challenge influenced by both genetic and environmental factors. The gut microbiota and its overall composition play an essential role during CRC initiation and progression, showing that an equilibrated microbial balance is essential in a healthy individual. This review highlights the specific organisms found in the gut microbiota, such as *Fusobacterium nucleatum* and *Bacteroides fragilis*, and how they strongly influence CRC risk and progression, with beneficial species and SCFAs providing protective effects. Some limitations, such as reliance on preclinical studies as well as incomplete human data, highlight the need for further investigation to validate such strategies. Various preventative measures, such as increased dietary fiber intake, probiotics, prebiotics, and their combined outcomes under synbiotics, show great promise in reducing CRC risk. Additionally, FMT represents a novel therapeutic intervention with the potential to restore gut microbiota balance and decrease inflammation. Further research is necessary to elucidate the mechanism of action by which gut microbiota influences CRC progression. Such results could be used to create new preventative strategies. Specifically, future research should focus on understanding the interactions of different bacterial species in the human GI system and the host immune response, the long-term effects of FMT and varying results based on administration routes, as well as developing personalized dietary interventions according to a person’s individual risk profile. Doing so will enhance our understanding of cancer prevention and CRC treatments, leading to better patient outcomes.

## Abbreviations

CRC: colorectal cancer
FMT: fecal microbiota transplantation
SCFAs: short-chain fatty acids

## Declarations

### Copyright

© The Author(s) 2025.
